# Nonmotor Symptoms of Parkinson's Disease

**DOI:** 10.4061/2011/351461

**Published:** 2012-03-22

**Authors:** Irena Rektorova, Dag Aarsland, K. Ray Chaudhuri, Antonio P. Strafella

**Affiliations:** ^1^Movement Disorders Center, First Department of Neurology, Medical Faculty, St. Anne's University Hospital and Applied Neuroscience Research Group, Central European Institute of Technology CEITEC, Masaryk University, 625 00 Brno, Czech Republic; ^2^Department of Neurobiology, Care Sciences and Society, Alzheimer's Disease Research Center, Karolinska Institute, 141 86 Stockholm, Sweden; ^3^Centre for Age-Related Medicine, Stavanger University Hospital and Institute of Clinical Medicine, Akershus University Hospital, University of Oslo, 4005 Stavanger, Norway; ^4^National Parkinson Foundation Centre of Excellence, King's College Hospital, King's College, London SE5 9RS, UK; ^5^Movement Disorder Unit and E. J. Safra Parkinson Disease Program, Toronto Western Hospital, UHN and Imaging Research Centre, Centre for Addiction and Mental Health, University of Toronto, Toronto, ON, Canada M5T 2S8

 The nonmotor symptoms (NMSs) of Parkinson's disease (PD) have received a lot of attention in the last few years. Despite this fact, they have still been underrecognized and undertreated [1, 2]. NMS may include cognitive problems, apathy, depression, anxiety, hallucinations, and psychosis as well as sleep disorders, fatigue, autonomic dysfunction, sensory problems, and pain [1]. Since these symptoms substantially contribute to patients' quality of life and are a frequent cause of hospitalization and institutionalization, the NMSs and their management have been recognized by the UK National Institute for Clinical Excellence as an important unmet need in PD [3]. Besides dopamine and Lewy-type pathology involving both striatal and extrastriatal brain regions, deficits in other neurotransmitter systems and/or other types of brain pathologies seem to play a key role in the pathogenesis of NMS in PD [4–6].

 It has been clearly shown that dopaminergic treatment improves motor symptoms and the quality of life in patients with PD. However, it only partly improves some features of the NMS, and it is not free from nonmotor side effects such as hallucinations and other psychotic symptoms, irritability, sleep attacks, and impulse control disorders. Furthermore, one study found that the patients who survived at 20 years from PD diagnosis suffered mainly from the nondopaminergic symptoms including falls, choking, dysarthria, but also dementia, visual hallucinations, daytime somnolence, symptomatic postural hypotension, and urinary incontinence [7]. Therefore, the management of these non-dopaminergic symptoms is a priority for research in the near future.

 In addition to pharmacotherapy, high-frequency deep brain stimulation of the subthalamic nucleus (STN DBS) is a powerful surgical treatment in well-selected candidates with advanced PD. STN DBS leads to improvements in dopaminergic drug-sensitive symptoms and reductions in subsequent drug dose and dyskinesias [8]. Although quality of life improves substantially, the procedure cannot be recommended for the treatment of NMS, and it may even cause specific cognitive side effects and may increase the risk for suicide. Skilled speech and physical therapy with cueing to improve gait, cognitive therapy to improve transfers, exercises to improve balance, and training to build up muscle power and increase joint mobility are efficacious. Regular physical and mental exercise is therefore recommended for all PD patients [9].

 As outlined above, multidisciplinary approach including both pharmacological and nonpharmacological treatment of PD is essential; however, the current delivery of allied healthcare services is inadequate, and many people who require such care are not being referred to the relevant specialist. Parkinson's Standards of Care Consensus Statement will soon be released by the European Parkinson's Disease Association and should be implemented in Europe in the near future.

 Besides symptomatic treatment, search for the disease-modifying, neuroprotective, and restorative treatment of PD is ongoing. Despite many so far unresolved issues, gene- and stem-cell-based therapies as well as immunotherapy targeting alpha-synuclein might become treatment options in the future. Development of these therapies is dependent on an accurate and comprehensive understanding of the pathogenesis and pathophysiology of PD and on the ability of very early diagnosis of premotor stages of PD. Therefore, a search for specific biomarkers (clinical, neuroimaging, biochemical, genetic) for early (premotor) diagnosis and for the disease progression is essential and large multicenter trials are underway.

 This special issue is dedicated to nonmotor symptoms of PD and focuses on the above-mentioned “old-new” topics. We sincerely hope that it will provide readers with interesting new data as well as with comprehensive up-to-date reviews. We wish our readers pleasant and inspiring reading.

*Irena Rektorova* *Irena Rektorova* *Dag Aarsland* *Dag Aarsland* *K. Ray Chaudhuri* *K. Ray Chaudhuri* *Antonio P. Strafella* *Antonio P. Strafella*
